# Analysis of Wastewater Samples to Explore Community Substance Use in the United States: Pilot Correlative and Machine Learning Study

**DOI:** 10.2196/45353

**Published:** 2023-10-26

**Authors:** Marie A Severson, Sathaporn Onanong, Alexandra Dolezal, Shannon L Bartelt-Hunt, Daniel D Snow, Lisa M McFadden

**Affiliations:** 1 Division of Basic Biomedical Sciences University of South Dakota Vermillion, SD United States; 2 Water Sciences Laboratory & Nebraska Water Center, part of the Daugherty Water for Food Global Institute University of Nebraska-Lincoln Lincoln, NE United States; 3 Department of Civil and Environmental Engineering University of Nebraska-Lincoln Lincoln, NE United States

**Keywords:** methamphetamine, opioids, substance use disorder, wastewater-based surveillance, drug detection, pilot study, substance use, detecting, monitoring, drugs, surveillance, community

## Abstract

**Background:**

Substance use disorder and associated deaths have increased in the United States, but methods for detecting and monitoring substance use using rapid and unbiased techniques are lacking. Wastewater-based surveillance is a cost-effective method for monitoring community drug use. However, the examination of the results often focuses on descriptive analysis.

**Objective:**

The objective of this study was to explore community substance use in the United States by analyzing wastewater samples. Geographic differences and commonalities of substance use were explored.

**Methods:**

Wastewater was sampled across the United States (n=12). Selected drugs with misuse potential, prescriptions, and over-the-counter drugs and their metabolites were tested across geographic locations for 7 days. Methods used included wastewater assessment of substances and metabolites paired with machine learning, specifically discriminant analysis and cluster analysis, to explore similarities and differences in wastewater measures.

**Results:**

Geographic variations in the wastewater drug or metabolite levels were found. Results revealed a higher use of methamphetamine (z=–2.27, *P*=.02) and opioids-to-methadone ratios (oxycodone-to-methadone: z=–1.95, *P*=.05; hydrocodone-to-methadone: z=–1.95, *P*=.05) in states west of the Mississippi River compared to the east. Discriminant analysis suggested temazepam and methadone were significant predictors of geographical locations. Precision, sensitivity, specificity, and *F*_1_-scores were 0.88, 1, 0.80, and 0.93, respectively. Finally, cluster analysis revealed similarities in substance use among communities.

**Conclusions:**

These findings suggest that wastewater-based surveillance has the potential to become an effective form of surveillance for substance use. Further, advanced analytical techniques may help uncover geographical patterns and detect communities with similar needs for resources to address substance use disorders. Using automated analytics, these advanced surveillance techniques may help communities develop timely, tailored treatment and prevention efforts.

## Introduction

Substance use disorder (SUD) is a widespread, debilitating disorder with extensive impacts, including social stress, economic impact, and death [1,2]. Considerable variability exists across the United States in measures of substance use, such as drug overdose mortality and survey results [3-5]. Additionally, treatment for SUD depends on the type of drug being used and its accessibility in communities. Consider the logistics of obtaining methadone, a prescription medication used to treat opioid use disorder (OUD) [6]. The median travel time to an opioid treatment program was 61 minutes in rural locations versus 12 minutes in urban locations [7]. Patients who had to travel more than 10 miles were also significantly more likely to miss methadone treatment doses compared to those closer to the treatment program [8]. The development of tools to help capture the impact of these health disparities is needed. To address communities’ specific needs for treating SUDs effectively, large-scale monitoring of substance use or misuse in the population using cost-efficient, noninvasive, rapid, and unbiased methods is required [9,10].

In the United States, methods of monitoring substance use include surveys or emergency room visits reported by the Substance Abuse and Mental Health Services Administration (SAMHSA) [5] or overdose mortality data from the Centers for Disease Control [3,4]. These methods provide essential information about SUD, but they are not without limitations. Surveys require respondents to reveal personal information about sensitive topics such as illicit activities, leading to fear and hesitation to respond honestly, thus potentially generating biased results [9,10]. Often, it requires the individual to be contacted via phone, email, or at a household, thus under-sampling people with unstable housing and those without access to phones or the internet. Indeed, homelessness is common among those experiencing SUD, with 1 study finding that over 40% of participants with SUD were homeless [11]. Overdose data also have limitations. It is highly influenced by the purity of drugs or the addition of contaminants such as fentanyl [3,12]. Similarly, assessing overdose mortality may be complicated by inconsistent use of standardized language and codes, and variations in postmortem toxicology testing by jurisdiction, which in turn may contribute to the underreporting of drug-related deaths [13,14]. Finally, hospitalizations or deaths due to substance use represent the worst-case scenario. Developing methods to detect substance use in communities in near real-time such that early interventions can be deployed is critically needed.

Although wastewater-based surveillance has gained much attention in recent years due to its ability to detect SARS-CoV-2 infections in a population [15,16], it has not yet been adopted for widespread drug detection and monitoring in the United States [9,17]. One objective of this study was to use wastewater surveillance methods that could be implemented in a wide variety of communities, including those that are resource-limited. Specifically, this study sought to assess community substance use from wastewater samples. Moreover, wastewater-based surveillance is just the first step toward improving public health responses to address SUD in rural and underserved communities as well as urban areas. Prior research suggests that wastewater may provide better predictive modeling and lead time when paired with machine learning compared to more traditional surveillance methods [18], highlighting the need to explore the implementation of machine learning for other wastewater surveillance methods. Applying advanced analysis techniques can offer additional insight into unique and common aspects of community substance use. Understanding these patterns can allow for more targeted and personalized public health policy and treatment strategies to be implemented. Therefore, this study tested the ability of machine learning, specifically discriminant analysis and hierarchical cluster analysis, to produce algorithms from wastewater-based surveillance data that could generalize to real-world drug use measures. With continued widescale monitoring, machine learning of wastewater results may help communities reduce SUD and use strategies tailored to their unique needs.

## Methods

### Ethical Considerations

Ethical guidelines for sewage surveillance to monitor drug use set forth by Prichard et al [19] were followed. To reduce the potential that the communities served by the wastewater plants would be negatively impacted by the findings, plants have been deidentified. To further reduce the risk of identifying communities, we report population rates as suggested by Prichard et al [19]. Only the broad geographical location (west or east of the Mississippi River) is given. These efforts align with ethical guidelines for wastewater-based surveillance set forth by the Sewage Analysis Core group Europe to help minimize risk to participating communities and their citizens [20]. The University of South Dakota’s Institutional Research Board determined the study to not meet the definition of human participant research because data in this project is not identifiable.

### Wastewater Samples

Staff from wastewater plants were recruited throughout the United States via email. Upon agreement to participate, plant supervisors from 12 wastewater plants were sent a survey of the plant characteristics, the population served, and sampling materials.

Polar Organic Chemical Integrative Samplers (POCIS; Environmental Sampling Technologies) were placed into the inlet of 12 wastewater plants for 7 days in March using methods similar to those previously described [21]. This sampling strategy was used to allow diverse populations to participate and has been previously used to quantify drugs in wastewater [21-25]. POCIS loaded with 3 Oasis hydrophilic-lipophilic balance sorbent were mounted within a perforated stainless-steel deployment canister. At each sampling location, the canister was secured in the sewage intake immediately after the grit chamber, before any chemical treatment took place, and it remained there for 1 week [21]. The canister was retrieved, rinsed with water, and then packed on ice and shipped to the University of Nebraska Water Sciences Laboratory in Lincoln, Nebraska. Samplers were stored at –20 °C until analysis.

### Analysis of Drugs and Metabolites

Upon receiving samples from all participating communities, drugs and their metabolites were extracted from each hydrophilic-lipophilic balance sorbent according to prior research [21], resulting in triplicate results for each treatment plant. Before extraction, each POCIS device was disassembled, and the hydrophilic-lipophilic balance sorbent was carefully transferred through gravity-flow chromatography columns. Approximately 10 mL of methanol was used to rinse any remaining sorbent from the membrane into the chromatography column. Target compounds were eluted from the sorbent using 50 mL of 1:1 methanol or acetonitrile with 0.1% ammonium hydroxide, and slowly passed through the resin into glass evaporation tubes (RapidVap N2, Labconco). Then, 80 μL of 1 ng/μL surrogate recovery standards (D11-amphetamine, orphenadrine, and ^13^C_3_-deethylatrazine) were added and mixed with each extract, which was then evaporated under nitrogen at 40 °C to approximately 1 mL. The sampler extract was quantitatively transferred to glass culture tubes using additional methanol and spiked with isotope-labeled internal standards (250 µL of carbamazepine-^13^C_6_, methamphetamine-d8, MDMA-d5, and MDA-d5, sulfamethazine-^13^C_6_, caffeine-^13^C_3_, morphine-d3, methadone-d3, oxycodone-d6, hydrocodone-d6, and temazepam-d5). The spiked extract was evaporated under nitrogen to a final volume of 80 µL and then mixed with 320 µL of 10 mM ammonium formate in water before transferring into an autosampler vial containing a 300 µL silanized glass insert. Standards for drug compounds, labeled surrogates, and internal standards were purchased from Sigma-Aldrich or Cerilliant.

Drugs and metabolites were quantified using an Agilent 6410 triple quadrupole mass spectrometer with electrospray ionization interfaced with an 1100 high-performance liquid chromatography (HPLC) system. Gradient separation was carried out using a Hypurity C18 reverse phase HPLC column (250 mm × 2.1 mm × 5 µm particle size) at a temperature of 50 °C and a flow rate of 0.2 mL/minute. Electrospray ionization was carried out in a positive mode. MS capillary voltage was 4000 V, the gas flow was 12 L/minute, and nebulizer pressure was 40 psi. Mass transitions, fragmentor voltages, collision energies, and instrument detection limits of each compound are given in Table 1.

POCIS extracts were analyzed for 1,7–dimethylxanthine (caffeine metabolite), acetaminophen (nonsteroidal anti-inflammatory drug), caffeine (a nonregulated stimulant), cotinine (nicotine metabolite), 3,4-methylenedioxymethamphetamine (MDMA, psychostimulant), methamphetamine (METH, psychostimulant), morphine (opioid pain reliever), hydrocodone (opioid pain reliever), methadone (medication used to treat OUD), oxycodone (opioid pain reliever), and temazepam (short-term sleep aid, benzodiazepine). Time-weighted average (*C*_w_) concentrations of the individual compounds in sampled wastewater were estimated by:


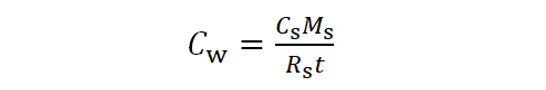
 (1)

where *C*_s_ is the concentration of the compound in the sorbent phases, *M*_s_ is the mass of sorbent, and *t* is the exposure time [21]. Sampling rates, *R*_s_ (L/d), were used from previously published studies (see Table 2 for values). Collective excretion rates (CER) were calculated by the following formula [22]:

CER=*C*_w_*Q* (2)

where *Q* is the plant flow rate (L/day×g/10^9^ ng). The CER per capita was calculated by dividing the CER by the population served and converting g to µg. The population served was determined by information reported by the wastewater treatment plant.

**Table 1:** Quantification characteristics of analytes.

Analyte	Parent ion (m/z)	Product ion (m/z)	Fragmentor (V)	Collision energy (eV)	Instrument detection limit (pg)
1,7-Dimethylxanthine	181.0	124.0	90	20	1.74
Acetaminophen	152.0	110.0	90	15	2.51
Caffeine	195.0	138.0	110	20	4.24
Cotinine	177.0	98.0	90	20	4.01
MDMA^a^	194.0	163.0	80	8	1.88
METH^b^	150.0	91.0	80	20	2.37
Morphine	286.0	165.0	150	40	8.13
Hydrocodone	300.2	199.0	168	26	4.23
Metaxalone	222.1	161.1	105	7	3.16
Methadone	310.2	265.1	115	11	1.66
Oxycodone	316.2	298.1	148	15	3.79
Temazepam	301.1	255.1	122	20	1.67

^a^MDMA: 3,4-methylenedioxymethamphetamine.

^b^METH: methamphetamine.

**Table 2:** Sampling rates (Rs) for each analyte found in the literature were used to calculate observed concentrations of analyte found in wastewater. Time-weighted average (Cw; ng/L).

Analyte	Sampling rate (*R*_s_)	Source	*C*_w_ (ng/L), median (IQR)
1,7-Dimethylxanthine	0.046	Bartelt-Hunt et al [23]	848.41 (572.09-2148.04)
Acetaminophen	0.048	Bartelt-Hunt et al [23]	2449.80 (1363.87-7337.70)
Caffeine	0.044	Bartelt-Hunt et al [23]	19 078.02 (9424.05-29,977.54)
Cotinine	0.034	Bartelt-Hunt et al [23]	259.51 (128.79-563.98)
MDMA^a^	0.222	Yargeau et al [24]	2.41 (0.24-4.14)
METH^b^	0.231	Yargeau et al [24]	63.03 (25.27-181.71)
Morphine	0.261	Yargeau et al [24]	11.36 (7.65-40.80)
Hydrocodone	0.050	Alvarez (personal communication)	43.09 (19.66-74.61)
Methadone	0.408	Yargeau et al [24]	5.27 (1.49-12.49)
Oxycodone	0.152	Yargeau et al [24]	13.25 (6.36-19.02)
Temazepam	0.421	MacLeod et al [25]	8.14 (3.95-17.97)

^a^MDMA: 3,4-methylenedioxymethamphetamine.

^b^METH: methamphetamine.

### Statistical Analysis

Samples were analyzed in triplicates (3 per location) and averaged. SAS Studio was used for all analyses. Spearman correlation coefficient was used to assess correlations among drugs or metabolites. Briefly, the CER for each analyte was correlated. Mann-Whitney *U* was used to test for differences in drug or metabolite CER levels per capita (CER/pop) between regions (east and west), given violations in normality. The ratio of opioid-to-methadone (opioid/methadone) was also tested using Mann-Whitney U.

Wastewater treatment plants were classified by geographical location using the Mississippi River as a division between east and west. Of note, this study used samples collected from only the contiguous United States. Determining unique patterns of drug consumption in different geographical areas is critical for developing targeted interventions and prevention campaigns. Therefore, linear discriminant analysis [26] was used to determine which analytes (1,7–dimethylxanthine, acetaminophen, caffeine, cotinine, MDMA, METH, morphine, hydrocodone, methadone, oxycodone, and temazepam) uniquely represent drug consumption in the east and west. This was chosen due to its performance with a smaller sample size and its ability to perform with nonnormally distributed data. Analyte values were included as the independent variables, and geographical location was considered the dependent variable. Stepwise selection was used in the linear discriminant analysis to determine which analytes best predict each geographical region. Briefly, forward selection was used to select a variable with the most discriminatory power in the model as Wilk λ. Backward elimination is then used to determine the variable with the least discriminatory power as measured by Wilk λ. The process of adding variables with high discriminatory power to the model and eliminating those with low discriminatory power continued until no more variables could be added or removed based on discriminatory power. The significance level to enter and to stay was set at a *P* value of .15. This was followed by leave-one-out cross-validation to assess the performance of the algorithm.

Hierarchical cluster analysis was also performed to assess the common and unique aspects of substance use in the communities served. Specifically, range standardized values were computed for cotinine CER/pop, MDMA CER/pop, methamphetamine CER/pop, morphine CER/pop, hydrocodone CER/pop, methadone CER/pop, oxycodone CER/pop, and temazepam CER/pop. Proc cluster was used to build dendrograms using the previously described variables using a Ward minimum-variance clustering method [27]. Briefly, cluster distance was determined by the sum of squares from the ANOVA between the 2 clusters added up over all of the variables. See documentation [27] for more information.

## Results

To better understand the relationship between substance use in the communities, the CERs were correlated with other analytes (see Table 3 for statistics). The opioids were correlated with other opioids and temazepam. There was also a significant correlation between the opioids analyzed and methadone. Interestingly, methadone was correlated with temazepam. METH was significantly correlated with hydrocodone, methadone, oxycodone, and temazepam. Cotinine was significantly correlated with other licit substances such as 1,7-dimethylxanthine, acetaminophen, and caffeine, as well as hydrocodone. As expected, 1,7-dimethylxanthine was significantly correlated with caffeine, acetaminophen, and cotinine. Acetaminophen was also significantly correlated with caffeine and MDMA. MDMA was significantly correlated with morphine. Finally, caffeine was also significantly correlated with hydrocodone.

**Figure 1:**
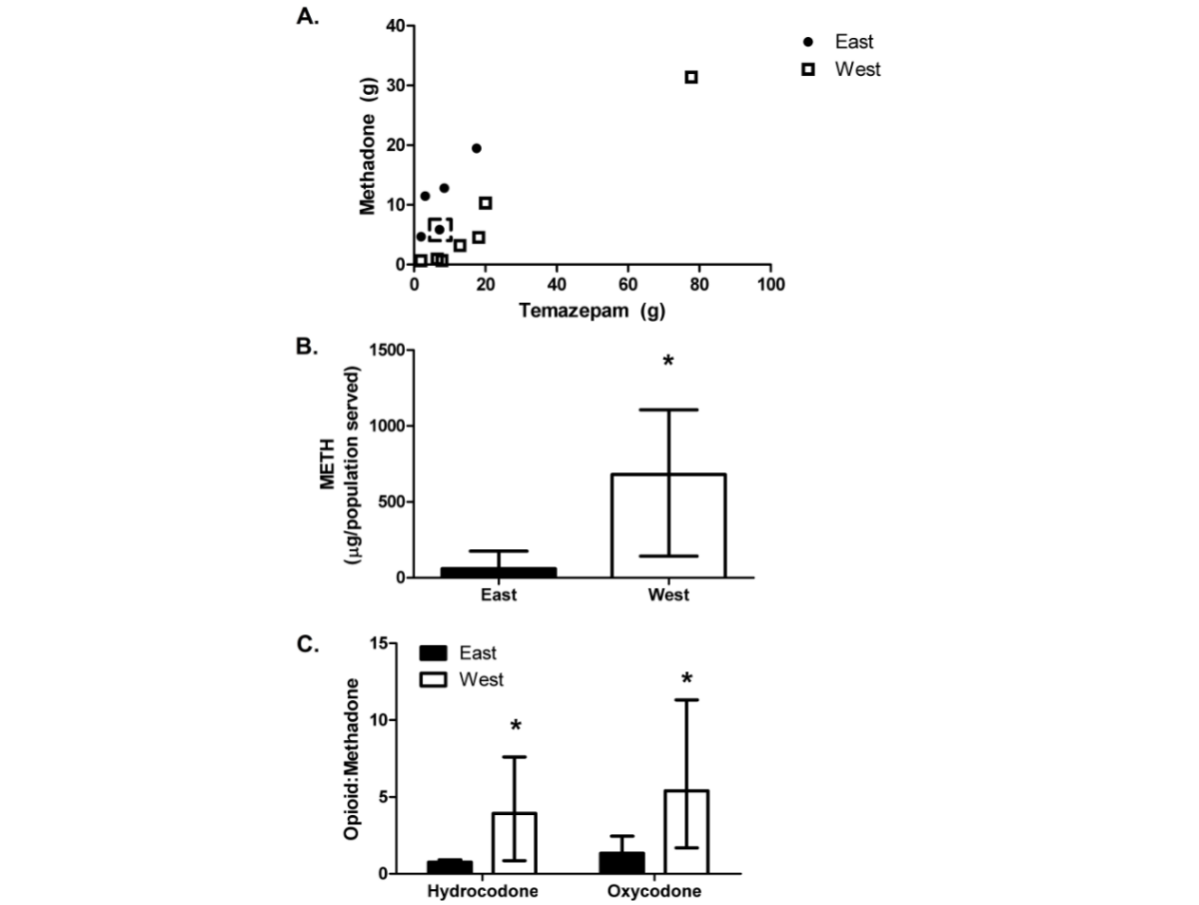
Patterns of drug use across geographical regions. (A) Linear discriminant analysis revealed that areas west of the Mississippi River (N=7) had higher temazepam levels compared to methadone whereas, in areas east of the river (N=5), the opposite pattern occurred. The wastewater plant that was misclassified is indicated by the dashed box. (B) Concentrations of METH were higher in wastewater plants west of the Mississippi River compared to the east (*P=.02). (C) Finally, the ratio of prescription opioids-to-methadone was examined. A higher ratio was found in wastewater in the west compared to the east (*P=.05); Bars represent the median (IQR); west versus east. METH: methamphetamine.

Differences in regional drug consumption were assessed. To account for differences in the population served, first, CERs of analytes were normalized to population (CER divided by population served). Cities west of the Mississippi River had higher METH levels compared to cities east of the river (Figure 1; z=–2.27, *P*=.02). Although the western cities had higher METH use, no other analyte differed significantly between the regions (Table 4).

Next, to assess the availability of methadone, a therapy for OUD, the ratio of the CER for an opioid analyte to the methadone analyte was assessed. The ratio of oxycodone-to-methadone (Figure 1; z=–1.95, *P*=.05) and hydrocodone-to-methadone (z=–1.95, *P*=.05) were significantly higher in the west compared to the east. This suggests less use of methadone compared to the opioids used in the western part of the United States. However, no difference was noted in the ratio of morphine-to-methadone between the regions (z=0.33, *P*=.74).

To determine what patterns are most representative of drug use in areas east and west of the Mississippi River, a linear discriminant analysis was completed. Stepwise selection was used to find the best variables that predict the geographic regions, resulting in the selection of methadone and temazepam (Methadone: Wilk λ= 0.50, *P*=.04; Temazepam: Wilk λ= 0.24, *P*=.008; Figure 1). The east was associated with higher methadone concentrations in sampled water and lower temazepam levels compared to the west. These variables correctly predicted the geographic location of 92% of the wastewater plants. Only 1 eastern plant was misclassified when cross-validation of the algorithm was performed, resulting in 100% of the western plants being correctly classified and 80% of the eastern plants. Precision, sensitivity, specificity, and F1 scores were 0.88, 1, 0.80, and 0.93, respectively.

**Figure 2:**
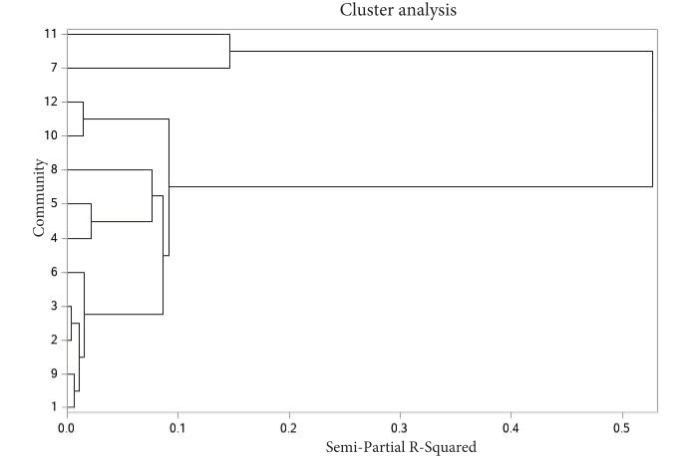
Cluster analysis of wastewater levels. Wastewater levels of drugs with the potential for misuse were clustered to assess communities with similar profiles.

Cluster analysis was also used to create a dendrogram to help determine which communities might benefit from working together to address SUDs (Figure 2). Results revealed that communities 7 and 11 formed 1 cluster disparate from the other communities. This cluster had a semipartial *R*^2^ value of 0.16. These 2 communities had the highest levels of drugs or metabolites in their wastewater, suggesting that the need for interventions may be high. The cluster formed by communities 4, 5, and 8 (semipartial *R*^2^=0.08) represented areas with high opioid and moderate METH levels. The remaining communities had lower levels of drugs in their wastewater. These findings highlight the variations of drug use in communities and the need to develop approaches to meet the community’s needs. With larger-scale monitoring, these clustering methods may be useful for helping communities with similar needs work together to develop targeted interventions.

**Table 3:** The correlation among analytes. Spearman rank correlation coefficient was used to correlate the various analytes.

Analyte and measured statistic		1,7-dimethyl-xanthine	Acetaminophen	Caffeine	Cotinine	MDMA^a^	METH^b^	Morphine	Hydrocodone	Methadone	Oxycodone	Temazepam
**1,7-d** **imethyl-xanthine**												
	ρ	—^c^	0.69	0.85	0.87	0.26	0.53	0.47	0.52	0.23	0.35	0.41
	*t* test (*df*)	—	3.03 (10)	5.17 (10)	5.69 (10)	0.86 (10)	1.98 (10)	1.68 (10)	1.95 (10)	0.75 (10)	1.18 (10)	1.43 (10)
	*P* value	—	.01	<.001	<.001	.41	.08	.12	.08	.47	.27	.18
**Acetaminophen**												
	ρ	0.69	—	0.66	0.59	0.58	0.52	0.44	0.44	0.31	0.36	0.40
	*t* test (*df*)	3.03 (10)	—	2.76 (10)	2.34 (10)	2.28 (10)	1.95 (10)	1.56 (10)	1.55 (10)	1.02 (10)	1.23 (10)	1.37 (10)
	*P* value	.01	—	.02	.04	.05	.08	.15	.15	.33	.25	.20
**Caffeine**												
	ρ	0.85	0.66	—	0.99	0.25	0.50	0.44	0.62	0.50	0.32	0.49
	*t* test (*df*)	5.17 (10)	2.76 (10)	—	18.71 (10)	0.80 (10)	1.81 (10)	1.54 (10)	2.47 (10)	1.81 (10)	1.07 (10)	1.78 (10)
	*P* value	.004	.02	—	<.001	.44	.10	.15	.03	.10	.31	.11
**Cotinine**												
	ρ	0.87	0.59	0.99	—	0.20	0.52	0.42	0.63	0.47	0.32	0.49
	*t* test (*df*)	5.69 (10)	2.34 (10)	18.71 (10)	—	0.63 (10)	1.91 (10)	1.46 (10)	2.56 (10)	1.68 (10)	1.07 (10)	1.78 (10)
	*P* value	.001	.04	<.001	—	.54	.08	.17	.03	.12	.31	.11
**MDMA**												
	ρ	0.26	0.58	0.25	0.20	—	0.26	0.60	0.45	0.41	0.41	0.50
	*t* test (*df*)	0.86 (10)	2.28 (10)	0.80 (10)	0.63 (10)	—	0.84 (10)	2.36 (10)	1.59 (10)	1.41 (10)	1.44 (10)	1.83 (10)
	*P* value	.41	.05	.44	.54	—	.42	.04	.14	.19	.18	.10
**METH**												
	ρ	0.53	0.52	0.50	0.52	0.26	—	0.47	0.85	0.64	0.83	0.85
	*t* test (*df*)	1.98 (10)	1.95 (10)	1.81 (10)	1.91 (10)	0.84 (10)	—	1.66 (10)	5.02 (10)	2.61 (10)	4.62 (10)	5.17 (10)
	*P* value	.08	.08	.10	.08	.42	—	.13	<.001	.03	.001	<.001
**Morphine**												
	ρ	0.47	0.44	0.44	0.42	0.60	0.47	—	0.75	0.71	0.75	0.70
	*t* test (*df*)	1.68 (10)	1.56 (10)	1.54 (10)	1.46 (10)	2.36 (10)	1.66 (10)	—	3.62 (10)	3.23 (10)	3.54 (10)	3.07 (10)
	*P* value	.12	.15	.15	.17	.04	.13	—	.005	.009	.005	.01
**Hydrocodone**												
	ρ	0.52	0.44	0.62	0.63	0.45	0.85	0.75	—	0.88	0.88	0.96
	*t* test (*df*)	1.95 (10)	1.55 (10)	2.47 (10)	2.56 (10)	1.59 (10)	5.02 (10)	3.62 (10)	—	5.89 (10)	5.89 (10)	10.57 (10)
	*P* value	.08	.15	.03	.03	.14	<.001	.005	—	<.001	<.001	<.001
**Methadone**												
	ρ	0.23	0.31	0.50	0.47	0.41	0.64	0.71	0.88	—	0.75	0.88
	*t* test (*df*)	0.75 (10)	1.02 (10)	1.81 (10)	1.68 (10)	1.41 (10)	2.61 (10)	3.23 (10)	5.89 (10)	—	3.57 (10)	5.89 (10)
	*P* value	.47	.33	.10	.12	.19	.03	.009	<.001	—	.005	<.001
**Oxycodone**												
	ρ	0.35	0.36	0.32	0.32	0.41	0.83	0.75	0.88	0.75	—	0.90
	*t* test (*df*)	1.18 (10)	1.23 (10)	1.07 (10)	1.07 (10)	1.44 (10)	4.62 (10)	3.54 (10)	5.89 (10)	3.57 (10)	—	6.35 (10)
	*P* value	.27	.25	.31	.31	.18	.001	.005	<.001	.005	—	<.001
**Temazepam**												
	ρ	0.41	0.40	0.49	0.49	0.50	0.85	0.70	0.96	0.88	0.90	—
	*t* test (*df*)	1.43 (10)	1.37 (10)	1.78 (10)	1.78 (10)	1.83 (10)	5.17 (10)	3.07 (10)	10.57 (10)	5.89 (10)	6.35 (10)	—
	*P* value	.18	.20	.11	.11	.10	<.001	.01	<.001	<.001	<.001	—

^a^MDMA: 3,4-methylenedioxymethamphetamine.

^b^METH: methamphetamine.

^c^—: Not available.

**Table 4:** Median analyte collective excretion rates values in the regions west and east of the Mississippi. Analyte values were normalized to the population served.

Analyte	Median analyte (µg) per population, median (IQR)			*z* score		*P* value
	East	West				
1,7-Dimethylxanthine	1912.55 (1038.03-3466.89)	8490.47 (1878.03-11,118.15)	–1.46		.14	
Acetaminophen	6804.48 (3071.81-7213.99)	11 903.19 (4463.67-53 219.64)	–0.81		.42	
Caffeine	48 595.92 (26,435.34-95,659.40)	85 838.70 (17,809.02-15,1082.80)	–0.49		.63	
Cotinine	902.48 (401.77-1250.37)	1894.11 (258.71-2268.31)	–0.81		.42	
MDMA^a^	0.43 (0.15-14.16)	10.57 (3.75-20.39)	–1.06		.29	
METH^b^	59.61 (25.61-175.27)	680.98 (141.47-1106.51)	–2.27		.02	
Morphine	25.85 (15.28-183.24)	58.01 (0.00-197.59)	0.00		>.99	
Hydrocodone	14.20 (5.80-38.21)	22.12 (9.58-57.71)	–0.97		.33	
Methadone	15.90 (11.18-56.63)	7.05 (2.32-73.92)	0.97		.33	
Oxycodone	30.29 (16.82-51.20)	57.84 (15.75-208.98)	–0.97		.33	
Temazepam	18.34 (4.35-44.26)	28.40 (17.11-183.08)	–0.97		.33	

^a^MDMA: 3,4-methylenedioxymethamphetamine.

^b^METH: methamphetamine.

## Discussion

### Principal Results

This study sought to use wastewater sampling paired with advanced computational methods to monitor substance use more rapidly across geographic areas. Using linear discriminant analysis, methadone and temazepam were identified as the 2 variables that best represent differences in the pattern of substance use east and west of the Mississippi River. Temazepam use was higher in the west compared to the east, whereas methadone was higher in the east compared to the west. When examining the ratio of opioid-to-methadone, plants west of the Mississippi River had higher opioid levels compared to methadone, suggesting access to this treatment may be lower than in areas in the east. Results also suggest there was greater METH use west of the Mississippi River. Overall, these findings suggest that this multimodal approach may be useful for revealing geographical differences in SUDs.

### Comparisons of Wastewater Sampling Techniques

These findings expand on the basic wastewater methodology established by previous studies. Many prior wastewater surveillance efforts in the United States have focused on specific brief occasions, such as sporting events [28,29]. While these studies have yielded interesting findings, these are unique events that may not generalize to daily living, thus limiting their use as a tool for public health surveillance. Using wastewater surveillance in the United States across multiple geographical areas over a longer duration is critical for developing public health surveillance tools that will allow for rapid responses. POCIS sampling, as opposed to active sampling, allows for more inclusive recruitment of communities. Hahn et al [30] reported that active sampling is costly and challenging to implement in limited-resource settings. Wastewater plants in rural communities and other areas with limited resources may not have the staff and other resources to participate in studies requiring daily sampling [31]. Capturing temporal variability in concentrations would require analysis of composite grab samples collected using either on-site staff (labor intensive and costly) or installation and operation of refrigerated automated wastewater samplers (technologically intensive and costly). While active sampling allows for capturing finer temporal variability, the cost and resources may be limiting factors. POCIS have many advantages over traditional grab sampling, including the lower cost for extended water monitoring periods between 7 and 30 days with the ability to estimate time-weighted average concentrations for compounds where uptake rates are published or measured. Recent studies comparing surface water grab sampling results to POCIS-derived time-weighted average concentrations differ by less than a factor of 5 for the majority (>80%) of pharmaceutical compounds monitored [32]. Pharmaceutical concentrations in wastewater treatment systems may vary considerably over time. In some cases, daily relative SDs may exceed 100% of the mean [33]. POCIS offer low cost, simple deployment, and good in situ preconcentration factors, which substantially outweigh the benefits of collecting and analyzing wastewater composite samples, especially in resource-limited settings.

SUDs are chronic diseases associated with a problematic pattern of use [34]. Because of this need to capture the chronic, cumulative drug use paired with the need to include resource-limited communities, POCIS sampling paired with limited surveys was used as opposed to daily sampling with extensive surveys. This allowed for the participation of wastewater plants from areas with limited resources. Beyond this inclusive sampling strategy, analytics were performed to assess geographical patterns of drug use.

### Comparison of Analysis Results to Prior Drug Surveillance Works

Within the United States, regional differences in METH use have been reported [35]. Consistent with the findings from this study, 70% of law enforcement agencies reporting from the West Coast and Midwest regions stated that METH was their greatest drug threat [36]. Both hospitalizations from METH-related poisoning and self-reported METH use are higher outside the Northeast area of the United States [5,37]. However, there are fewer facilities that will treat METH use disorder in western states when compared to states east of the river [38]. The results of this study align with methods traditionally used and suggest METH use is higher west of the Mississippi.

Further, this study confirms that opioid use was similar in wastewater west and east of the Mississippi River. Pain reliever use disorder and hospitalizations were largely similar among these geographical regions [5,37]. These results suggest that the analytical techniques used produced results that align with other validated methods of detecting substance use. However, the combination of wastewater and analytical methods used here has the potential to produce much more rapid and automated results than traditional methods.

The current techniques used also provide insights into treatments for OUD. Although methadone is a well-known treatment for opioid misuse, its use is limited by accessibility [39-41]. People from rural and small urban locations often cite transportation to treatment and distance to treatment as a barrier on their journey to recovery [42]. This may contribute to the difficult decision to relocate from their permanent address to seek treatment. Further, there are distinct geographical variations in the accessibility of methadone as a treatment option for OUD. Nearly twice as many methadone facilities exist east of the Mississippi River compared to the west [38]. The limited access to methadone treatment may set back the recovery of people with OUD, particularly in the western states. Indeed, the results of this study suggest that the ratio of prescription opioids-to-methadone is higher in the west, which may be mediated by fewer treatment facilities in the area. Further, methadone was a key predictor variable when linear discriminant analysis was used to determine the unique drug patterns between the east and west. These wastewater and analytical findings provide further evidence of the need for SUD treatment options in the western part of the United States.

Discriminant analysis results also suggested that temazepam was a significant predictor of geographical locations. This may be particularly problematic given polysubstance drug use and overdoses have increased over the past decade [14,37]. Benzodiazepines are commonly misused by people who experience OUD and were involved in 21.7% of all opioid overdose deaths [43-45]. In this study, temazepam was highly correlated with the opioids analyzed. These significant correlations warrant further investigation to understand the extent and risks imposed by polysubstance drug use. Together, the results of this study highlight unique patterns of drug use that can be derived from geographical locations. Determining unique patterns of drug consumption in different geographical areas is critical for developing targeted interventions.

### Developing Monitoring Tools Using Wastewater and Analytics

Wastewater-based surveillance for community drug use monitoring allows for low-cost, rapid, and noninvasive testing of entire communities while filling the gaps in drug use surveys and other traditional drug use analyses [17]. Importantly, advanced machine learning analytics of wastewater-based surveillance (discriminant analysis) results can be used to detect substance use trends in specific geographic areas, even with relatively few data points. Further, they can help identify communities with similar patterns of use. Unsupervised machine learning (cluster analysis) was used to cluster communities based on the unique combinations of analytes found in wastewater. This helps identify communities with similar needs for SUD treatments. By clustering these communities, implementation plans to address SUD needs can be efficiently developed while avoiding a one-size-fits-all strategy. Through clustering, distinct groups of communities with high drug use overall, high opioid use and moderate methamphetamine use, and lower overall drug use were found. Not only can wastewater surveillance be used as an early indicator of changing drug use patterns, but it can also strengthen the conclusions drawn from traditional methods [46].

Wastewater-based surveillance, when paired with further analysis, can help provide more timely information to elucidate a more complete picture of substance use in the United States. Given rapid increases in drug overdoses during the COVID-19 pandemic, the advantages of wastewater surveillance for substance use may be critical for navigating the rapidly changing situation [47]. Although the sample size was limited in this study, results still replicated findings derived from more traditional surveillance methods. As large-scale wastewater surveillance is being implemented for monitoring SARS-CoV-2 [48], the methods used in sample collecting and machine learning, specifically discriminant analysis and hierarchical cluster analysis, can easily be scaled to expand existing wastewater surveillance efforts to monitor drug epidemics. Successes for this paired approach have been noted in the literature. For example, recent studies used random forests machine learning to predict hospitalizations from COVID-19 [18]. Using wastewater surveillance and machine learning, this model outperformed more traditional metrics, such as reported cases paired with machine learning, in predicting weekly hospital admissions, resulting in longer lead time and lower error rates. Expanding this infrastructure to include the surveillance of substances could help advance the monitoring of substance use. With greater implementation of wastewater surveillance for drug use, more information about community substance use can be derived, especially from populations with limited inclusions in traditional surveillance methods [49]. The inclusion of a greater population served by the facility is a benefit of this surveillance strategy compared to sampling a subset of residents, as occurs with traditional surveys that may not represent the entire population. Additionally, surveillance can include a variety of targeted compounds, such as prescription, over-the-counter, and illicit drugs. Similarly, emerging drugs not captured by surveys can be included. Finally, expanding advanced analytics of the resulting wastewater findings may provide critical information for communities to work together and develop the tools necessary to meet their unique needs for SUD treatment and prevention.

### Limitations

Volkow et al [50] called for comprehensive and timely data to address the substance use epidemic without a blindfold. Specifically, innovative data collection methods are needed to yield timely information on drug use inclusive of populations not normally included in traditional surveillance [50]. While digital surveillance of SUD has been increasing [51-55], other novel methods are needed to aid those with limited access to the internet. In this study, a linear discriminant analysis algorithm both performed with a smaller data set and generalized to real-world findings. The methods employed led to a sampling strategy in near real-time and inclusive of low-resource areas, enabling the surveillance of populations often missing or under-sampled in traditional drug surveillance efforts. The algorithm employed was also able to capture similar trends as those derived from conventional surveillance that require a much larger sample size. This is in part because each wastewater sample is a composite sample of the population served by the plant, thus more representative of the entire community than a single individual. However, more complicated algorithms are available, but they often require a much larger sample size, which would be both costly and require more time to process wastewater. The small sample sizes and less complex algorithms are limitations of this study. Other limitations of this study are related to wastewater surveillance. This form of surveillance does not capture information on individual users. Information on the individual user can be helpful for the development of personalized treatment plans for substance use. While this does not provide information on the individual users, which is a limitation, it does protect individuals’ privacy. Another limitation is that some individuals are served by septic systems instead of wastewater treatment plants. While this is true, this population may be captured when they visit more urban centers for school, work, or shopping. Finally, there are multiple ways to collect wastewater samples. While multiple composite or grab samples may provide better temporal resolution, POCIS sampling offers a lower cost and less labor-intensive sampling method, which is critical for developing inclusive drug surveillance. Further, the spatial resolution is limited to the population served by the wastewater treatment plant. However, higher spatial resolution could be obtained by sampling from sewer mains. For more information on analytical techniques used to analyze wastewater and the benefits and challenges associated with these methods and wastewater surveillance, please see Hahn et al [30], Huizer et al [56], and Erickson et al [57]. With the combination of inclusive sampling methods plus advanced analysis, this study provides formative evidence that these methods can be generalized in near real-time and deployed in real-world population health settings.

### Future Studies

The findings of this study provided proof of concept that wastewater surveillance and advanced analytical techniques can be combined to monitor substances. While it offered foundational knowledge, future studies are needed. Notably, including a greater number of sampling locations and longer durations will allow for a fuller understanding of similarities and differences in community drug use. This will enable more complex forms of machine learning to be used. Similar to techniques used in monitoring COVID-19, these models may provide critical predictions on future health care use related to substance use, which may help communities prepare and proactively address issues as they arise. Future studies may pair these results with community interventions to address substance use. Although beyond the scope of this study, it may provide valuable information about the current challenges a community is facing as well as the short and long-term changes associated with interventions trialed by the community. Finally, continued monitoring can allow for assessing emerging drug threats that can help inform people who use substances and health care providers of potential contaminants in the drug supply and other emerging threats. For example, the linear discriminant analysis used in this study could also identify communities with a new contaminant in the drug supply. Communities with this impurity could receive resources to reduce the harm associated with the contaminant. In contrast, communities without the contaminant could receive training on what to expect if this enters their drug supply. This would allow communities facing similar needs to work together while avoiding a one-size-fits-all strategy.

### Conclusions

Wastewater surveillance has become increasingly used to monitor population health [16,58]. Advanced analytical techniques, specifically discriminant and hierarchical cluster analyses, may further the utility of wastewater surveillance to help discover patterns and clusters of substance use that would otherwise be overlooked and speed up the time it takes to reveal patterns. In this study, wastewater surveillance was successfully used in multiple municipalities across the United States to provide insights into regional drug use patterns. We used prior traditional surveillance methods such as surveys by SAMHSA, reports by law enforcement, CDC findings, and other research previously published to help validate these findings [5,14,35-41,43-45]. With the help of discriminant analysis, findings indicated less access to methadone treatment in states west of the Mississippi River and higher methamphetamine levels. Further, wastewater clustering analysis of levels of substances with the potential for misuse revealed community clusters with high, moderate, and low levels of substance use. Although these findings are preliminary and cannot be extrapolated to all communities in the United States, with greater use of both wastewater-based surveillance and advanced analytical techniques, the resulting information could provide valuable insights into identifying communities most in need of targeted SUD treatments and prevention programs in a more rapid and cost-efficient manner.

## Abbreviations

CER: collective excretion rates
HPLC: high-performance liquid chromatography
MDMA: 3,4-methylenedioxymethamphetamine
METH: methamphetamine
OUD: opioid use disorder
POCIS: Polar Organic Chemical Integrative Samplers
SAMHSA: Substance Abuse and Mental Health Services Administration
SUD: substance use disorder

## Data Availability

Wastewater data for the study are not publicly available due to community privacy reasons but are available from the corresponding author upon reasonable request.
